# MUC1-C Oncoprotein Regulates Glycolysis and Pyruvate Kinase m2 Activity in Cancer Cells

**DOI:** 10.1371/journal.pone.0028234

**Published:** 2011-11-28

**Authors:** Michio Kosugi, Rehan Ahmad, Maroof Alam, Yasumitsu Uchida, Donald Kufe

**Affiliations:** Dana-Farber Cancer Institute, Harvard Medical School, Boston, Massachusetts, United States of America; University of Texas Health Science Center at Houston, United States of America

## Abstract

Aerobic glycolysis in cancer cells is regulated by multiple effectors that include Akt and pyruvate kinase M2 (PKM2). Mucin 1 (MUC1) is a heterodimeric glycoprotein that is aberrantly overexpressed by human breast and other carcinomas. Here we show that transformation of rat fibroblasts by the oncogenic MUC1-C subunit is associated with Akt-mediated increases in glucose uptake and lactate production, consistent with the stimulation of glycolysis. The results also demonstrate that the MUC1-C cytoplasmic domain binds directly to PKM2 at the B- and C-domains. Interaction between the MUC1-C cytoplasmic domain Cys-3 and the PKM2 C-domain Cys-474 was found to stimulate PKM2 activity. Conversely, epidermal growth factor receptor (EGFR)-mediated phosphorylation of the MUC1-C cytoplasmic domain on Tyr-46 conferred binding to PKM2 Lys-433 and inhibited PKM2 activity. In human breast cancer cells, silencing MUC1-C was associated with decreases in glucose uptake and lactate production, confirming involvement of MUC1-C in the regulation of glycolysis. In addition, EGFR-mediated phosphorylation of MUC1-C in breast cancer cells was associated with decreases in PKM2 activity. These findings indicate that the MUC1-C subunit regulates glycolysis and that this response is conferred in part by PKM2. Thus, the overexpression of MUC1-C oncoprotein in diverse human carcinomas could be of importance to the Warburg effect of aerobic glycolysis.

## Introduction

Cancer cells are distinguished from their normal counterparts by metabolic differences that include increased utilization of glucose by aerobic glycolysis. This characteristic of malignant cells, known as the Warburg effect, associates a high rate of glucose consumption with enhanced lactate production in the presence of oxygen [1]. Aerobic glycolysis in cancer cells has been linked with increased expression of glycolytic genes [2], [3]. Pyruvate kinase (PK) is one of the upregulated glycolytic gene products that catalyzes the production of pyruvate and ATP from phosphoenolpyruvate (PEP) and ADP. There are four PK isoenzymes, M1, M2, L and R. The M1 isoform is expressed in most adult cells. The M2 isoform (PKM2), a splice variant of M1, is found in embryonic cells, certain normal proliferating cells and cancer cells [4]. Heterogenous nuclear ribonucleoproteins bind to PKM1 mRNA and inhibit its splicing [5]. Converting PKM2 expression to PKM1 in cancer cells reverses the Warburg effect and is associated with loss of tumorigenicity, establishing the importance of PKM2 for aerobic glycolysis and the proliferation of malignant cells [6]. The distinct region of PKM2, as compared to PKM1, functions in the allosteric activation of the enzyme by fructose-1,6-bisphosphate (FBP) [7] and its inactivation by phosphotyrosine containing proteins [8], [9]. Under these circumstances, the regulation of PKM2 activity dictates the metabolism of glucose to pyruvate, which is converted by lactate dehydrogenase (LDH) to lactate or is utilized by the mitochondrial tricarboxylic acid (TCA) cycle [10]. These findings have thus supported the need to more fully understand the signals that regulate aerobic glycolysis and PKM2 activity in malignant cells.

The mucin 1 (MUC1) protein is overexpressed in most human carcinomas and certain hematologic malignancies, making it one of the more common alterations in human cancers [11]. MUC1 is expressed as two subunits that form a heterodimer at the cell membrane. The large MUC1 N-terminal subunit (MUC1-N) is positioned extracellularly and contains the glycosylated tandem repeats that are characteristic of the mucin family. The MUC1 C-terminal subunit (MUC1-C) spans the cell membrane and contains a 58 amino acid extracellular domain and a 72 amino acid cytoplamic domain [11]. The MUC1-C extracellular domain interacts with galectin-3 and thereby forms complexes at the cell surface with the epidermal growth factor receptor (EGFR) [12]. Activation of EGFR is in turn associated with phosphorylation of the MUC1-C cytoplasmic domain [11]. Significantly, overexpression of the MUC1-C subunit, and specifically the cytoplasmic domain, is sufficient to induce anchorage-independent growth and tumorigenicity [13], [14]. With overexpression of MUC1 in cancer cells, the MUC1-C subunit accumulates in the cytoplasm and is targeted to the nucleus, where it contributes to the regulation of gene expression [11]. In this regard, MUC1-C-induced transformation is associated with the activation of genes involved with proliferation and tumorigenesis [15], [16]. MUC1-C also induces a signature associated with lipid metabolism and the upregulation of genes that regulate cholesterol and fatty acid synthesis [17]. Other studies have demonstrated that MUC1-C activates the PI3K->Akt pathway [18], [19], which in turn stimulates activity of the glycolytic enzymes, hexokinase and phosphofructose kinase. There is, however, no known link between MUC1-C and the glycolytic pathway.

The present studies demonstrate that MUC1-C is involved in the regulation of glucose uptake and lactate production in MUC1-C-induced transformation of rat fibroblasts and in human breast cancer cells. The results also demonstrate that the MUC1-C cytoplasmic domain interacts directly with PKM2 and regulates PKM2 activity. The MUC1-C cytoplasmic domain contains a Cys residue that binds to the PKM2 C-domain Cys-474 and stimulates PKM2 activity. By contrast, the EGFR-phosphorylated MUC1-C cytoplasmic domain interacts with the PKM2 C-domain at Lys-433 and inhibits PKM2. These findings indicate that the overexpression of MUC1-C in cancer cells contributes to the regulation of aerobic glycolysis.

## Results

### MUC1-C-induced transformation is associated with the induction of aerobic glycolysis

**Figure 1:**
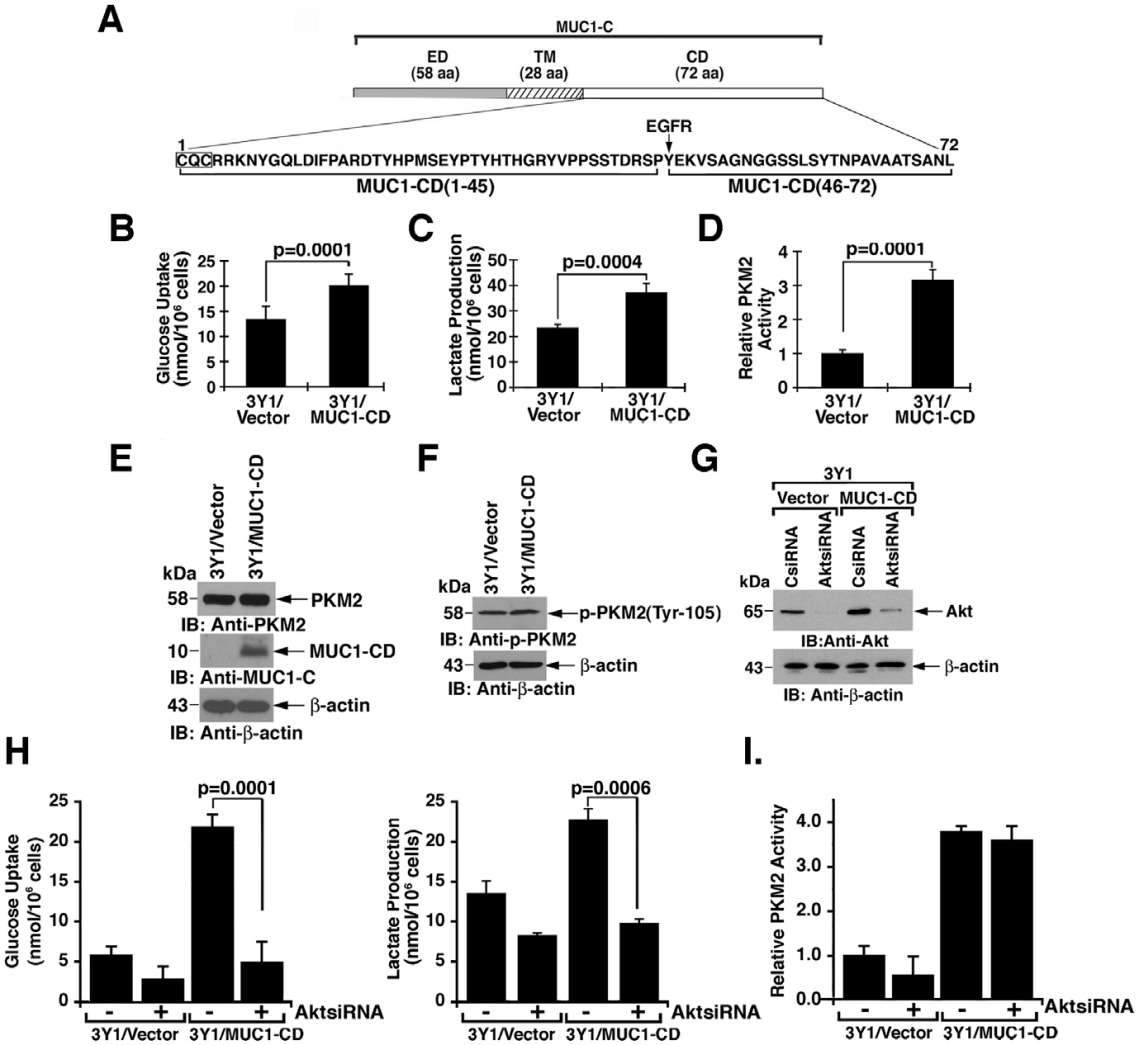
MUC1-C cytoplasmic domain (MUC1-CD)-induced transformation of 3Y1 cells is associated with induction of glycolysis and PKM2 activity. A. Structure of the MUC1-C subunit with the extracellular domain (ED), transmembrane domain (TM) and amino acid sequence of the cytoplasmic domain (MUC1-CD). The CQC motif and the EGFR phosphorylation site are highlighted. B and C. 3Y1/vector and 3Y1/MUC1-CD cells were analyzed for glucose uptake (B) and lactate production (C). The results (mean±SD of five separate experiments each performed in triplicate) are expressed as nmol/10^6^ cells. D. Lysates from rat 3Y1/vector and 3Y1/MUC1-CD cells were analyzed for PKM2 activity. The results (mean±SD of three separate experiments each performed in triplicate) are expressed as relative PKM2 activity compared to that obtained in 3Y1/vector cells (assigned a value of 1). The student's t-test was used to determine the p-values. E and F. Lysates from 3Y1/vector and 3Y1/MUC1-CD cells were immunoblotted with the indicated antibodies. G-I. 3Y1/vector and 3Y1/MUC1-CD cells were transfected with Control siRNA or Akt siRNA pools for 72 h. Lysates from the indicated cells were immunoblotted with anti-Akt and anti-β-actin (G). The indicated cells were analyzed for glucose uptake (H, left) and lactate production (H, right). The results (mean±SD of three replicates) are expressed as nmol/10^6^ cells. Lysates from the indicated cells were also analyzed for PKM2 activity (I). The results (mean±SD of three replicates) are expressed as relative PKM2 activity compared to that obtained in 3Y1/vector cells (assigned a value of 1).

The MUC1-C subunit consists of a 58 amino acid (aa) extracellular domain, a 28 aa transmembrane domain and a 72 aa cytoplasmic domain (Fig. 1A). Expression of the MUC1-C cytoplasmic domain (MUC1-CD) in 3Y1 fibroblasts is associated with the induction of colony formation in soft agar and tumor formation in nude mice [13], [14]. In the present studies, we found that MUC1-CD-induced transformation of 3Y1 cells is associated with an increase in glucose uptake (Fig. 1B) and lactate production (Fig. 1C), consistent with the stimulation of glycolysis. To assess, at least in part, the basis for this response, studies were performed to determine effects on PKM2. Notably, there was a significant increase in PKM2 activity in the MUC1-CD transformed 3Y1 fibroblasts (Fig. 1D). 3Y1/vector cells express PKM2, but not PKM1, and MUC1-CD-induced transformation had no apparent effect on PKM2 levels (Fig. 1E). Other studies have shown that PKM2 activity is inhibited by phosphorylation on Tyr-105 in certain cancer cells [9]. MUC1-CD-induced transformation was not associated with a change in Tyr-105 phosphorylation (Fig. 1F). In addition, there was no apparent cellular redistribution of PKM2 as determined by confocal microscopy (Fig. S1). Previous studies have demonstrated that Akt activation as determined by phosphorylation at Ser-473 is increased in 3Y1/MUC1-CD cells as compared to that found in 3Y1/vector cells [18]. To assess whether the increase in p-Akt is responsible for the activation of PKM2, we silenced Akt in the 3Y1/vector and 3Y1/MUC1-CD cells (Fig. 1G) and measured glucose uptake, lactate production and PKM2 activity. The results demonstrate that silencing Akt decreases glucose uptake in both 3Y1/vector and 3Y1/MUC1-CD cells (Fig. 1H, left). Moreover, silencing Akt in the 3Y1/vector and 3Y1/MUC1-CD cells was associated with partial decreases in lactate production (Fig. 1H, right). By contrast, silencing Akt had little if any effect on PKM2 activity in the 3Y1/MUC1-CD cells (Fig. 1I), indicating that MUC1-CD-mediated induction of PKM2 is not dependent on Akt activation.

### MUC1-C cytoplasmic domain Cys-3 residue interacts with PKM2

**Figure 2:**
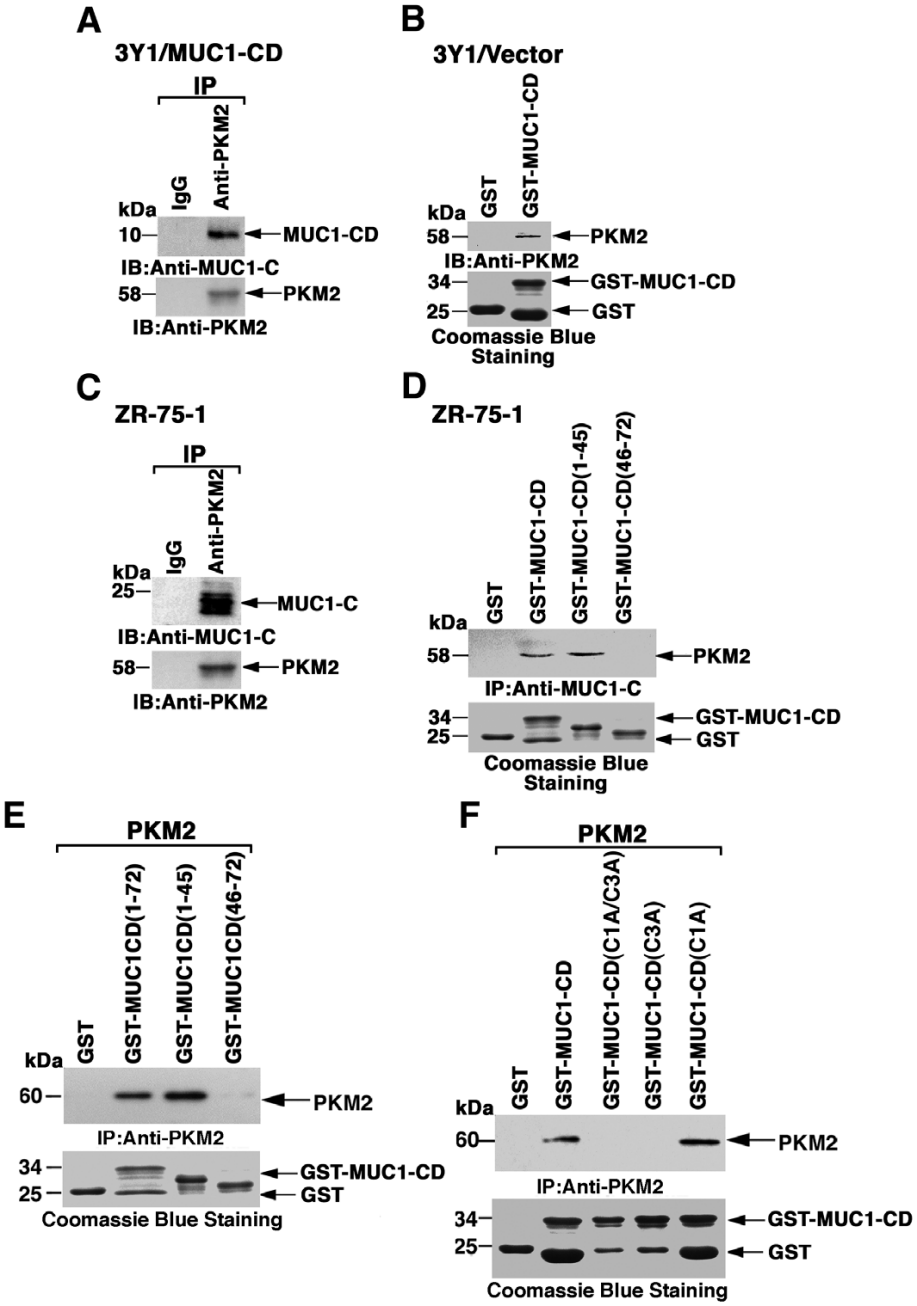
MUC1-C cytoplasmic domain binds to PKM2. A. Lysates from 3Y1/MUC1-CD cells were subjected to immunoprecipitation with a control IgG or anti-PKM2. The precipitates were immunoblotted with the indicated antibodies. B. Lysates from 3Y1/vector cells were incubated with GST or GST-MUC1-CD. The adsorbates to glutathione beads were immunoblotted with anti-PKM2. Input of the GST proteins was assessed by Coomassie blue staining. C. Lysates from human ZR-75-1 breast cancer cells were immunoprecipitated with a control IgG or anti-PKM2. Precipitates were immunoblotted with the indicated antibodies. D. ZR-75-1 cell lysates were incubated with GST, GST-MUC1-CD and the indicated GST-MUC1-CD deletion mutants. The adsorbates were immunoblotted with anti-PKM2. Input of the GST proteins was assessed by Coomassie blue staining. E. PKM2 was incubated with GST, GST-MUC1-CD and the indicated GST-MUC1-CD deletion mutants. The adsorbates were immunoblotted with anti-PKM2. F. PKM2 was incubated with GST, GST-MUC1-CD and the indicated GST-MUC1-CD mutants in which Cys-1 and/or Cys-3 were substituted with Ala. The adsorbates were immunoblotted with anti-PKM2.

To determine whether MUC1-CD interacts with PKM2 and thereby affects its activity, coimmunoprecipitation studies were performed with 3Y1/MUC1-CD cell lysates. Analysis of anti-PKM2 precipitates by immunoblotting with anti-MUC1-C supported the association of these proteins (Fig. 2A). In GST pull-down experiments, incubation of 3Y1/vector cell lysates with GST and GST-MUC1-CD provided further support for an interaction between PKM2 and MUC1-CD (Fig. 2B). To extend these observations to cells that express endogenous MUC1, coimmunoprecipitation studies were performed on lysates from human ZR-75-1 breast cancer cells. Here, PKM2 was detectable in complexes with the 25–20 kDa MUC1-C subunit (Fig. 2C). Pull-down experiments with ZR-75-1 cell lysates also demonstrated binding of MUC1-CD to PKM2 (Fig. 2D). Studies with MUC1-CD deletion mutants further showed that the association with PKM2 is conferred by MUC1-CD(1–45) and not MUC1-CD(46–72) (Fig. 2D). Incubation of GST-MUC1-CD with purified recombinant His-tagged PKM2 confirmed direct binding of MUC1-CD and PKM2 (Fig. 2E). The results also showed that MUC1-CD(1–45) and not MUC1-CD(46–72) is responsible for the direct interaction (Fig. 2E). MUC1-CD contains a CQC motif (aa 1–3) that contributes to the formation of MUC1-C homodimers (Fig. 1A) [20]. Mutation of the CQC motif to AQA (C1A/C3A) blocked binding of MUC1-CD to PKM2 (Fig. 2F). Binding of MUC1-CD to PKM2 was also abrogated with the C3A, and not the C1A, mutant, indicating that Cys-3 is responsible for the interaction (Fig. 2F). These findings indicate that MUC1-C associates with PKM2 in cells through a direct interaction mediated by the MUC1-C cytoplasmic domain Cys-3 residue.

### MUC1-CD binds directly to the PKM2 B-domain Cys-165 and C-domain Cys-474

**Figure 3:**
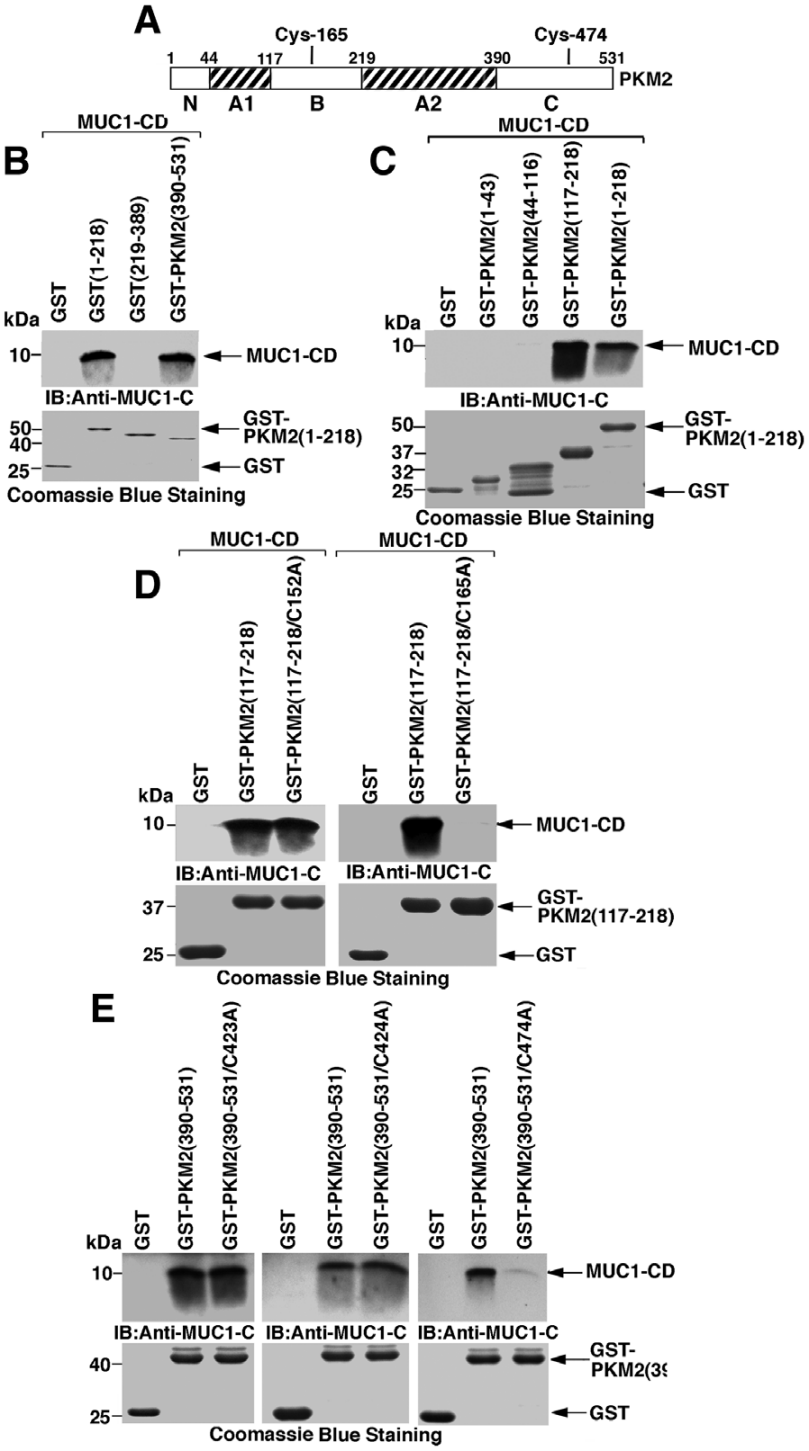
MUC1-CD binds directly to PKM2 B- and C-domains. A. Schematic representation of the PKM2 protein with the N-terminus (N) and the A1-, B-, A2- and C-domains. Highlighted are the Cys-165 and Cys-474 residues. B and C. MUC1-CD was incubated with GST and the indicated GST-PKM2 deletion mutants. The adsorbates were immunoblotted with anti-MUC1-C. Input of the GST proteins was assessed by Coomassie blue staining. D. MUC1-CD was incubated with GST, GST-PKM2(117-218) and GST-PKM2(117-218) with the indicated C152A and C165A mutations. The adsorbates were immunoblotted with anti-MUC1-C. E. MUC1-CD was incubated with GST, GST-PKM2(390-531) and GST-PKM2(390–531) with the indicated C423A, C424A and C474A mutations. The adsorbates were immunoblotted with anti-MUC1-C.

PKM2 consists of an N-terminus (aa 1–43), A1-domain (aa 44–116), B-domain (aa 117–218), A2-domain (aa 219–389) and C-domain (aa 390–531) [7] (Fig. 3A). Binding of MUC1-CD was detectable with PKM2(1–218) and PKM2(390–531), but not with PKM2(219–389) (Fig. 3B). Further analysis with PKM2(1–218) deletion mutants demonstrated that MUC1-CD binds directly to the B-domain (aa 117–218) and not the N-terminus (aa 1–43) or A1-domain (aa 44–116) (Fig. 3C). The PKM2 B-domain contains two cysteines at positions 152 and 165. Mutation of PKM2(117–218) Cys-152 to Ala (C152A) had no effect on the interaction with MUC1-CD (Fig. 3D, left). By contrast, binding of MUC1-CD and PKM2(117-218) was abrogated by mutating Cys-165 to Ala (C165A) (Fig. 3D, right). With regard to the PKM2 C-domain (aa 390–531), Cys residues are located at positions 423, 424 and 474. Binding of MUC1-CD to PKM2(390–531) was not affected by mutating Cys-423 to Ala (C423A) (Fig. 3E, left) or Cys-424 to Ala (C424A) (Fig. 3E, middle). However, the interaction with MUC1-CD was attenuated by mutation of the PKM2(390–531) Cys-474 residue to Ala (C474A) (Fig. 3E, right). These findings indicate that the MUC1-CD Cys-3 residue binds directly to (i) the PKM2 B-domain Cys-165, and (ii) the PKM2 C-domain Cys-474.

### Phosphorylated MUC1-CD binds to the PKM2 C-domain Lys-433

**Figure 4:**
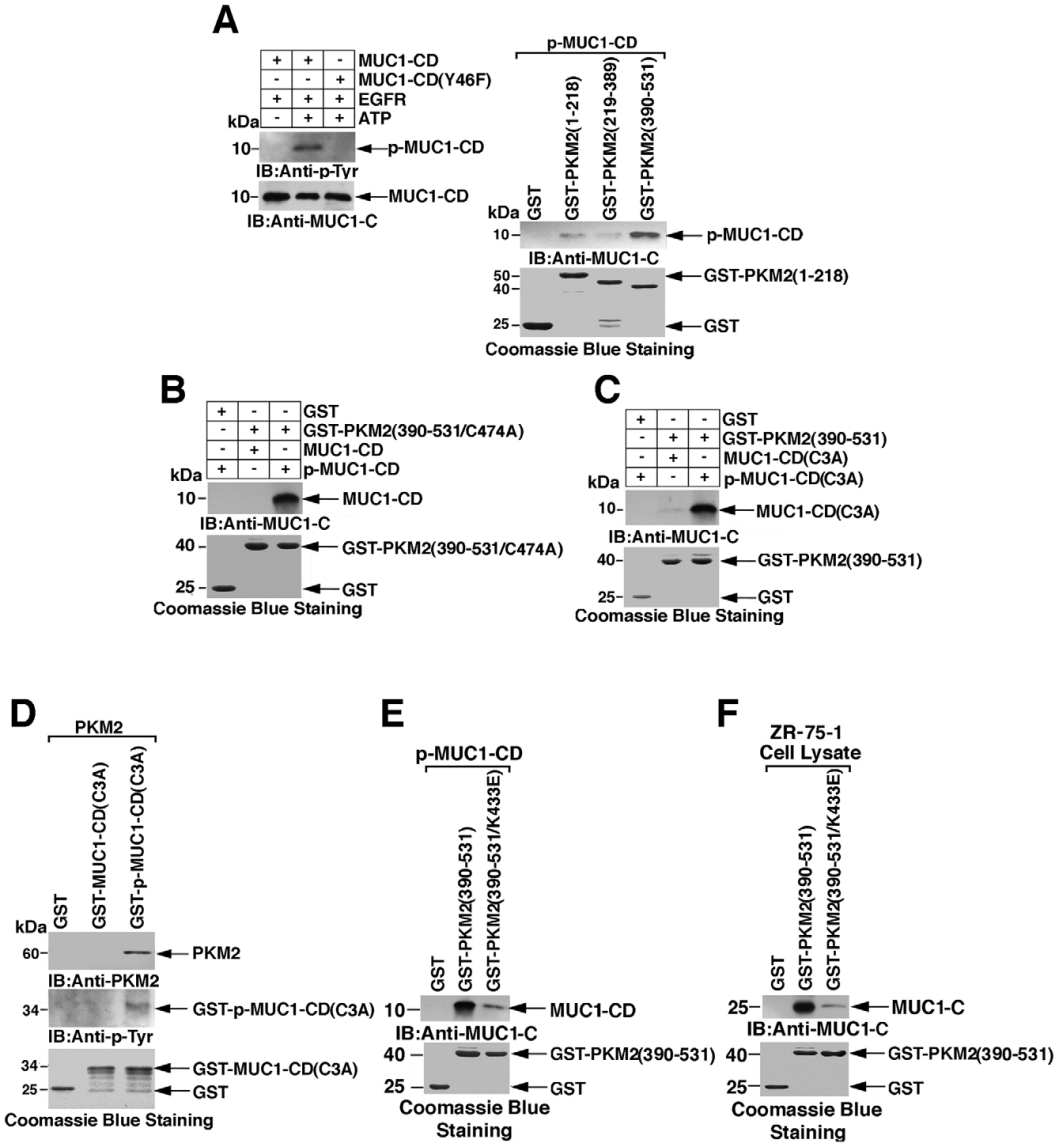
Phosphorylation of MUC1-CD on Tyr-46 confers binding to the PKM2 C-domain at Lys-433. A. His-tagged MUC1-CD and MUC1-CD(Y46F) were incubated with EGFR in the absence and presence of ATP. The reaction products were analyzed by immunoblotting with anti-p-Tyr and anti-MUC1-C (left). GST and the indicated GST-PKM2 deletion mutants were incubated with EGFR-phosphorylated MUC1-CD (p-MUC1-CD) (right). The adsorbates were immunoblotted with anti-MUC1-C. Input of the GST proteins was assessed by Coomassie blue staining. B. GST and GST-PKM2(390–531/C474A) were incubated with MUC1-CD or p-MUC1-CD. The adsorbates were immunoblotted with anti-MUC1-C. C. GST and GST-PKM2(390-531) were incubated with MUC1-CD(C3A) or EGFR-phosphorylated p-MUC1-CD(C3A). The adsorbates were immunoblotted with anti-MUC1-C. D. PKM2 was incubated with GST, GST-MUC1-CD(C3A) and EGFR-phosphorylated GST-p-MUC1-CD(C3A). The adsorbates were immunoblotted with anti-MUC1-C and anti-p-Tyr. E and F. GST, GST-PKM2(390-531) and GST-PKM2(390-531/K433E) were incubated with p-MUC1-CD (E) or ZR-75-1 cell lysate (F). The adsorbates were immunoblotted with anti-MUC1-C.

The MUC1-C cytoplasmic domain is phosphorylated on Tyr-46 by EGFR (Fig. 1A) [21]. Other studies have demonstrated that certain phosphotyrosine peptides interact with the PKM2 C-domain and inhibit its activity [8]. To determine whether tyrosine phosphorylated MUC1-C cytoplasmic domain binds to PKM2, we first incubated MUC1-CD and MUC1-CD(Y46F) with EGFR and ATP. Analysis of the reaction products by immunoblotting with anti-P-Tyr confirmed phosphorylation on Tyr-46 (Fig. 4A, left). As found with MUC1-CD, incubation of p-MUC1-CD with the PKM2 deletion mutants demonstrated binding to PKM2(1-218) and PKM2(390-531) (Fig. 4A, right). Binding of p-MUC1-CD was also detectable with PKM2(390-531/C474A) (Fig. 4B), supporting an interaction that is not mediated by the PKM2 Cys-474 residue. To extend these observations, the MUC1-CD(C3A) mutant, which is devoid of binding to PKM2, was subjected to EGFR phosphorylation. Comparison of MUC1-CD(C3A) and p-MUC1-CD(C3A) confirmed that EGFR phosphorylation induces binding to PKM2(390–531) (Fig. 4C). These results were also confirmed in experiments in which binding of full-length PKM2 was detectable with p-MUC1-CD(C3A) and not MUC1-CD(C3A) (Fig. 4D). The PKM2 C-domain contains a Lys-433 residue that is essential for phosphotyrosine peptide binding [8]. Indeed, mutation of PKM2(390–531) Lys-433 to glutamate (K433E) partially decreased binding of p-MUC1-CD (Fig. 4E). Incubation of ZR-75-1 cell lysates with GST-PKM2(390–531) and GST-PKM2(390–531/K433E) further demonstrated that binding to endogenous MUC1-C is partially decreased by the K433E mutation (Fig. 4F). These findings demonstrate that phosphorylation of the MUC1-C cytoplasmic domain Tyr-46 residue confers binding to the PKM2 C-domain at Lys-433.

### Stimulation of PKM2 activity by MUC1-CD Cys-3

**Figure 5:**
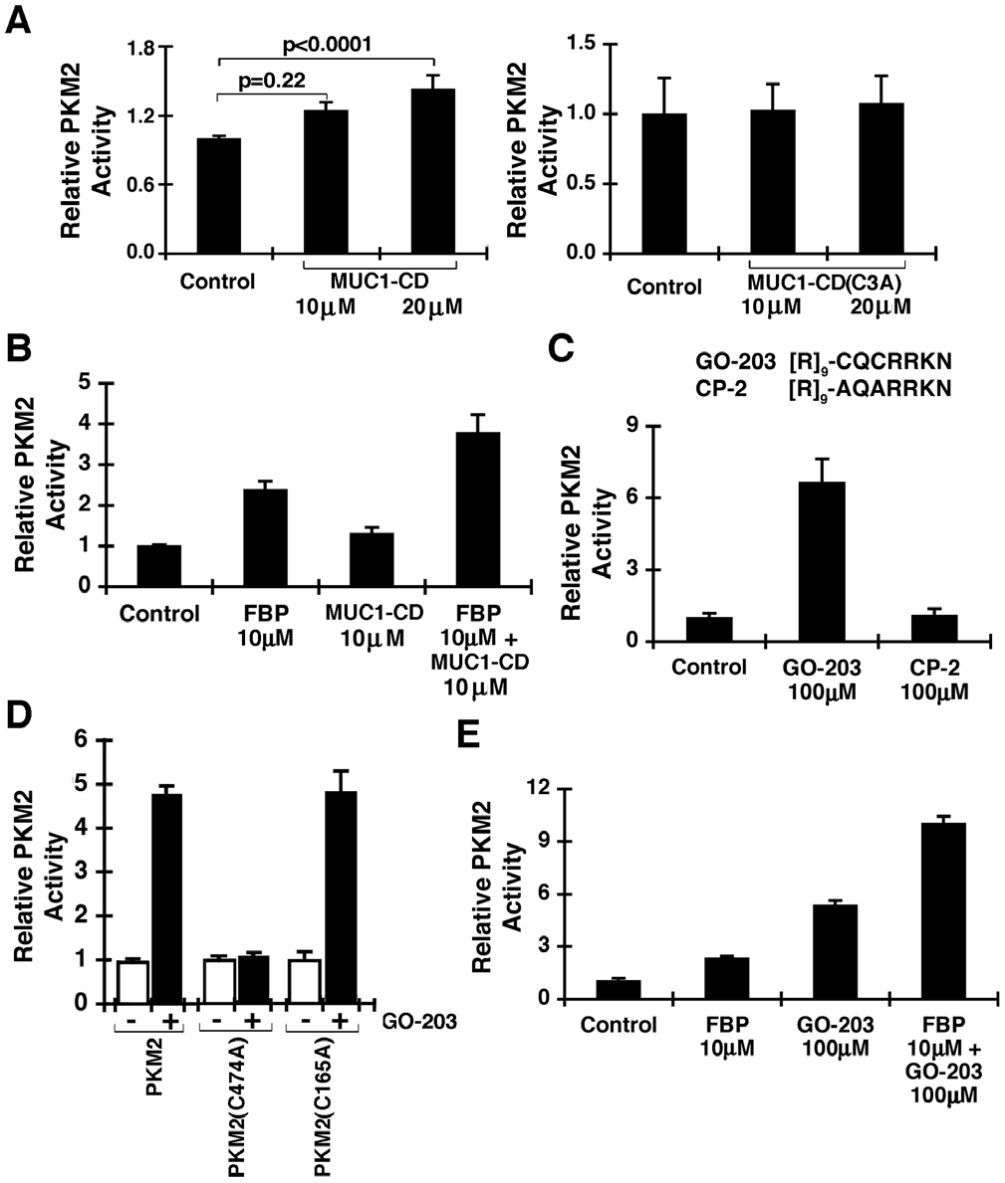
MUC1-CD CQC motif stimulates PKM2 activity. A. Recombinant PKM2 was incubated in the absence and presence of the indicated concentrations of MUC1-CD (left) or MUC1-CD (C3A) (right) for 30 min at room temperature. The results (mean+SD of five separate experiments) are expressed as the relative PKM2 activity compared to that obtained with the control. B. PKM2 was incubated with the indicated concentrations of FBP alone, MUC1-CD alone, or FBP and MUC1-CD. The results (mean+SD of three separate experiments) are expressed as the relative PKM2 activity compared to that obtained with the control. C. Amino acid sequences of GO-203 and CP-2 peptides. PKM2 was incubated with 100 µM GO-203 or CP-2. The results (mean+SD of five separate experiments) are expressed as the relative PKM2 activity compared to that obtained with the control. D. PKM2 and the indicated PKM2 mutants were incubated in the absence (open bars) and presence of 100 µM GO-203 (solid bars). The results (mean+SD of three separate experiments) are expressed as the relative PKM2 activity compared to that obtained in the absence of GO-203. E. PKM2 was incubated with the indicated concentrations of FBP alone, GO-203 alone, or FBP and GO-203. The results (mean+SD of three separate experiments) are expressed as the relative PKM2 activity compared to that obtained with the control.

PKM2 is allosterically activated by binding of FBP to the C-domain [7]. Of potential functional significance to the interaction between MUC1-CD and PKM2, incubation of MUC1-CD with PKM2 was associated with a modest stimulation of PKM2 activity (Fig. 5A, left). MUC1-CD-induced stimulation of PKM2 was dependent on the MUC1-CD Cys-3 motif in that MUC1-CD(C3A) had no apparent effect on PKM2 activity (Fig. 5A, right). As shown previously [7], FBP was effective in stimulating PKM2 activity (Fig. 5B). Moreover, the addition of both FBP and MUC1-CD resulted in at least an additive increase (Fig. 5B), indicating that MUC1-CD can promote FBP-induced PKM2 activation. To extend these observations, we incubated PKM2 with GO-203, a peptide that contains poly-Arg ([R]_9_) and the MUC1-CD sequence CQCRRKN containing the Cys-3 residue that was shown above to bind PKM2 (Fig. 5C). GO-203 was highly effective in stimulating PKM2 activity, whereas a control peptide, designated CP-2, in which the Cys-3 residue was substituted with Ala (AQARRKN) had little effect (Fig. 5C). Moreover, mutation of PKM2 Cys-474, but not Cys-165, to Ala resulted in abrogation of GO-203-induced increases in PKM2 activity (Fig. 5D). Incubation of PKM2 with both FBP and GO-203 further demonstrated that GO-203 is additive with FBP in inducing PKM2 activity (Fig. 5E). These findings indicate that the interaction between the MUC1-CD Cys-3 residue and PKM2 Cys-474 stimulates PKM2 activity.

### Tyrosine-phosphorylated MUC1-CD inhibits PKM2 activity

**Figure 6:**
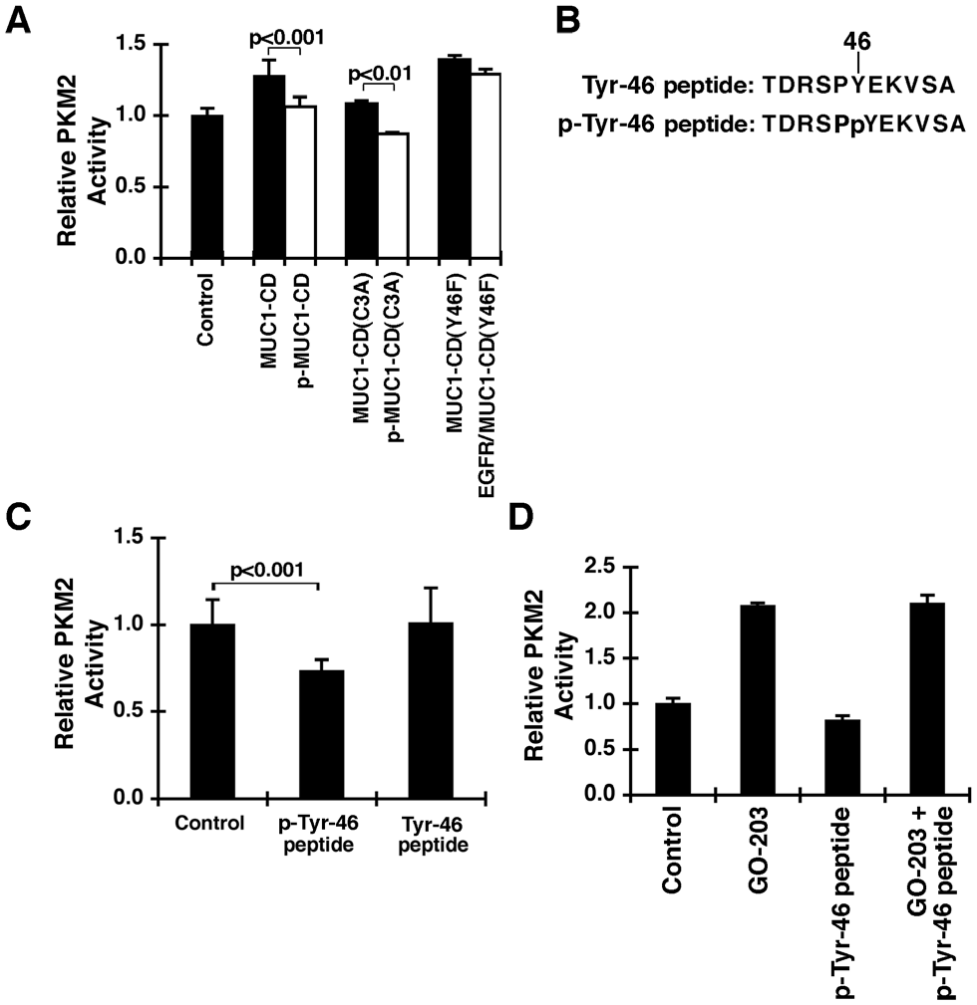
PKM2 activity is inhibited by EGFR-phosphorylated MUC1-CD. A. PKM2 was incubated with 10 µM FBP in the absence (Control) and presence of 10 µM unphosphorylated and EGFR-phosphorylated MUC1-CD, MUC1-CD(C3A) and MUC1-CD(Y46F). The results (mean+SD of three separate experiments) are expressed as the relative PKM2 activity compared to that obtained with the control. B. Sequences of the Tyr-46 and phospho-Tyr-46 peptides. C. PKM2 was incubated with 10 µM FBP in the absence (Control) and presence of 100 µM phospho-Tyr-46 peptide or Tyr-46 peptide. The results (mean+SD of three separate experiments) are expressed as the relative PKM2 activity compared to that obtained with the control. D. PKM2 was incubated in the absence (Control) and presence of 20 µM GO-203 alone, 100 µM phospho-Tyr-46 peptide alone or GO-203 with phospho-Tyr-46 peptide. The results (mean+SD of three separate experiments) are expressed as the relative PKM2 activity compared to that obtained with the control.

Binding of tyrosine-phosphorylated peptides to the PKM2 C-domain is associated with inhibition of PKM2 [8]. Consequently, we compared the effects of MUC1-CD and EGFR-phosphorylated p-MUC1-CD on PKM2 activity. As shown above, FBP-induced stimulation of PKM2 was increased by MUC1-CD (Fig. 6A). By comparison, phosphorylated p-MUC1-CD was ineffective in increasing PKM2 activity (Fig. 6A). These studies with p-MUC1-CD are, however, complicated by the potential for both stimulatory effects of Cys-3 and inhibitory effects of p-Tyr-46. Consequently, experiments were performed with the MUC1-CD(C3A) mutant, which is ineffective in stimulating PKM2. Here, MUC1-CD(C3A) had no apparent stimulatory effect and EGFR-phosphorylated MUC1-CD(C3A) suppressed PKM2 activity (Fig. 6A). As a control, the MUC1-CD(Y46F) mutant that had been incubated with EGFR and ATP had little if any effect (Fig. 6A). To confirm these observations, we synthesized peptides corresponding to the control and EGFR-phosphorylated MUC1-CD Tyr-46 (YEKV) motif (Fig. 6B). The phospho-Tyr-46 peptide, but not the unphosphorylated form, inhibited PKM2 activity (Fig. 6C). Experiments were also performed with GO-203 alone and in combination with the phospho-Tyr-46 peptide. Under these experimental conditions, GO-203-induced stimulation of PKM2 activity was unaffected by the phospho-Tyr-46 peptide (Fig. 6D). These findings indicate that EGFR-mediated phosphorylation of MUC1-CD on the YEKV motif inhibits PKM2 activity and that GO-203-induced stimulation of PKM2 is not blocked by this mechanism.

### MUC1-C promotes aerobic glycolysis in breast cancer cells

**Figure 7:**
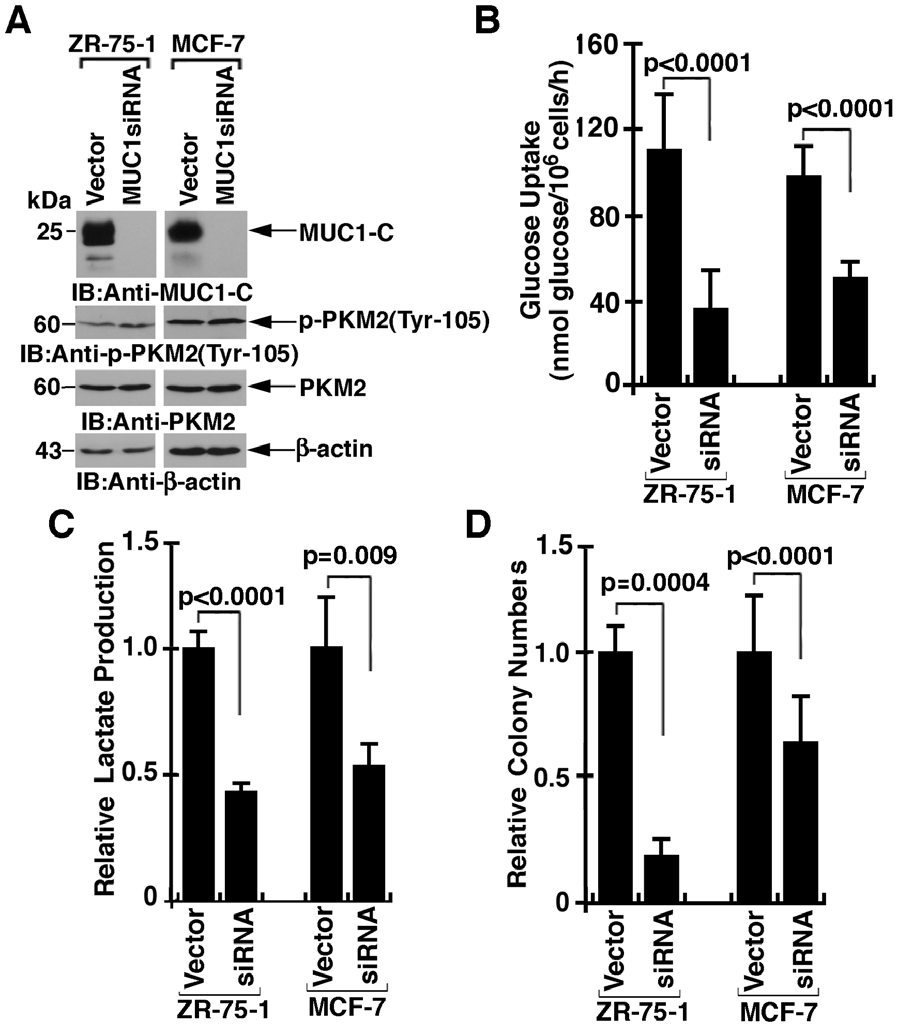
Silencing MUC1-C downregulates aerobic glycolysis in breast cancer cells. A. Lysates from ZR-75-1 and MCF-7 cells stably expressing an empty vector or a MUC1siRNA were immunoblotted with the indicated antibodies. B. The indicated cells were analyzed for glucose uptake. The results (mean±SD of three separate experiments each performed in triplicate) are expressed as nmol/10^6^ cells. C. The indicated cells were analyzed for lactate production. The results (mean±SD of three separate experiments each performed in triplicate) are expressed as relative lactate production compared to that in cells expressing the empty vector (assigned a value of 1). D. The indicated cells were plated in soft agar and colonies were counted after incubation for 14 days. The results (mean±SD of two separate experiments each performed in triplicate) are expressed as the colony number relative to that obtained with ZR-75-1 or MCF-7 cells expressing an empty vector (each assigned a value of 1).

To determine whether endogenous MUC1-C affects glycolysis, we studied ZR-75-1 and MCF-7 breast cancer cells with stable silencing of MUC1-C expression (Fig. 7A). Downregulation of MUC1-C had no effect on PKM2 levels or phosphorylation on Tyr-105 (Fig. 7A). Significantly, analysis of both ZR-75-1 and MCF-7 cells demonstrated that silencing MUC1-C is associated with decreased glucose uptake (Fig. 7B) and decreased lactate production (Fig. 7C), indicating that MUC1-C promotes glycolysis in breast cancer cells. In addition to the suppression of glycolysis, silencing MUC1-C conferred decreases in ZR-75-1 and MCF-7 colony formation (Fig. 7D).

### Effects of MUC1-C on PKM2 activity in breast cancer cells

**Figure 8:**
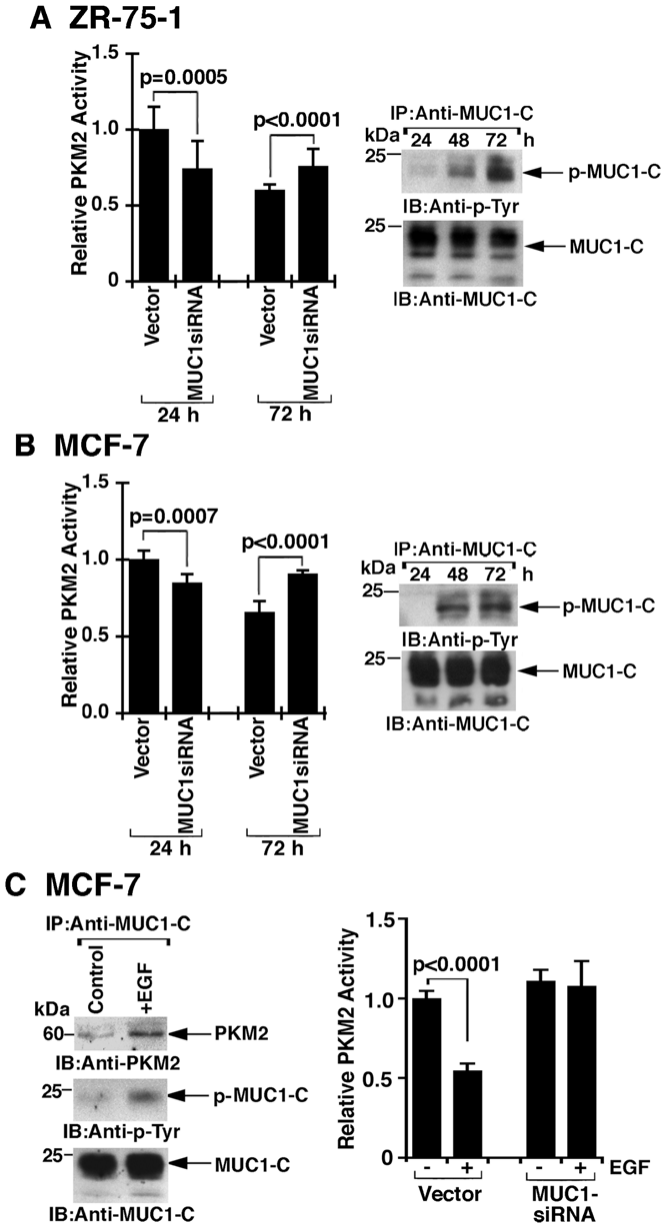
Effects of MUC1-C on PKM2 activity are dependent on cell context. A and B. Lysates from the indicated ZR-75-1 (A) and MCF-7 (B) cells harvested at the indicated times after passage were analyzed for PKM2 activity (left). The results (mean+SD of three separate experiments each performed in triplicate) are expressed as relative PKM2 activity compared to that obtained in cells expressing the empty vector (assigned a value of 1). The ZR-75-1/vector (A) and MCF-7/vector (B) cell lysates were also incubated with anti-MUC1-C and the precipitates were immunoblotted with the indicated antibodies (right). C. MCF-7/vector and MCF-7/MUC1siRNA cells were left untreated and stimulated with EGF for 5 min. Lysates from MCF-7/vector cells were incubated with anti-MUC1-C and the precipitates were immunoblotted with the indicated antibodies (left). Lysates were assayed for PKM2 activity (right). The results (mean+SD of three separate experiments) are expressed as the relative PKM2 activity as compared to that in unstimulated MCF-7/vector cells.

Studies were performed to determine whether the effect of MUC1-C on aerobic glycolysis in breast cancer cells is associated with changes in PKM2 activity. At 24 h after passage of ZR-75-1 cells, PKM2 activity was decreased with downregulation of MUC1-C expression (Fig. 8A, left). By contrast, at 72 h of culture, silencing MUC1-C was associated with an increase in PKM2 activity (Fig. 8A, left). These observations corresponded with an increase in the extent of MUC1-C tyrosine phosphorylation from 24 to 72 h of culture (Fig. 8A, right). Similar results were obtained with MCF-7 cells (Fig. 8B, left and right), indicating that the MUC1-C tyrosine phosphorylation status contributes to the regulation of PKM2. In that line of reasoning, studies were performed to assess the effects of EGF stimulation. Treatment of the MCF-7/vector cells with EGF was associated with an increase in phosphorylation of MUC1-C on tyrosine (Fig. 8C, left). Moreover, EGF stimulation of MCF-7/vector, but not MCF-7/MUC1siRNA, cells resulted in downregulation of PKM2 activity (Fig. 8C, right). These findings indicate that tyrosine phosphorylated MUC1-C suppresses PKM2 activity in breast cancer cells and that this effect is more pronounced in the response to EGF stimulation.

### Effects of expressing a MUC1(C3A) mutant on PKM2 activity

**Figure 9:**
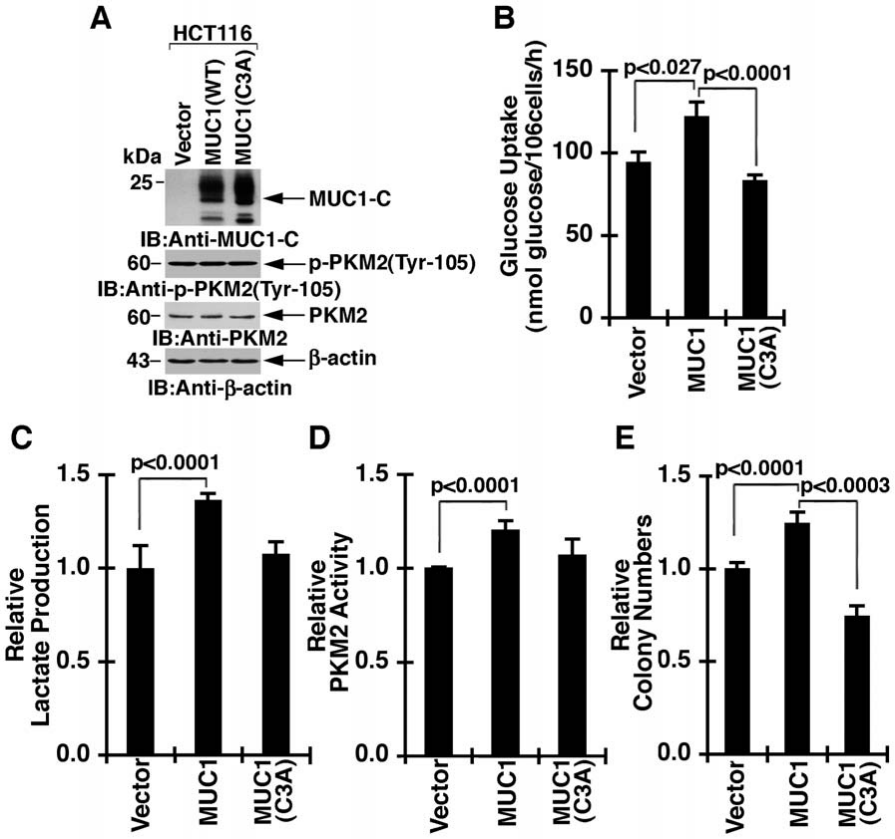
Effects of MUC1 on glycolysis and PKM2 activity are attenuated by the C3A mutation. A. Lysates from HCT116 cells stably expressing an empty vector, wild-type MUC1 or MUC1(C3A) were immunoblotted with the indicated antibodies. B. The indicated cells were analyzed for glucose uptake. The results (mean±SD of three separate experiments each performed in triplicate) are expressed as nmol/106 cells. C. The indicated cells were analyzed for lactate production. The results (mean±SD of three separate experiments each performed in triplicate) are expressed as relative lactate production compared to that in cells expressing the empty vector (assigned a value of 1). D. Lysates from the indicated cells were analyzed for PKM2 activity. The results (mean±SD of three separate experiments each performed in triplicate) are expressed as relative PKM2 activity compared to that obtained in cells expressing the empty vector (assigned a value of 1). E. The indicated cells were plated in soft agar and colonies were counted after incubation for 14 days. The results (mean±SD of two separate experiments each performed in triplicate) are expressed as the colony number relative to that obtained with HCT116/vector cells (assigned a value of 1).

To determine whether the effects of silencing MUC1-C could be attributed to the interaction between the MUC1-C cytoplasmic domain and PKM2, studies were performed with HCT116 cells, which are null for MUC1 expression and were stably transfected to express an empty vector, wild-type MUC1 or MUC1 with the C3A mutation (Fig. 9A). MUC1 and MUC1(C3A) expression had no effect on PKM2 or p-PKM2(Tyr-105) levels (Fig. 9A). Glucose uptake was increased in HCT116/MUC1, but not HCT116/MUC1(C3A), cells (Fig. 9B). Similar results were obtained when measuring lactate production (Fig. 9C). MUC1, but not MUC1(C3A), expression was also associated with increases in PKM2 activity (Fig. 9D). Moreover, as shown previously [20], MUC1 expression was associated with increases in HCT116 colony formation and this effect was abrogated by the MUC1(C3A) mutant (Fig. 9E). These findings indicate that blocking the interaction between MUC1-CD and PKM2 attenuates the MUC1-CD-mediated effects on glycolysis, PKM2 activity and colony formation.

## Discussion

### MUC1-C oncoprotein promotes glycolysis

Most cancer cells are dependent on aerobic glycolysis for the generation of energy that is needed for cellular processes. This altered metabolism, known as the Warburg effect, involves increased uptake of glucose with decreased utilization of the TCA cycle, such that pyruvate generated during glycolysis is converted to lactate [22]. This metabolic switch, which generates intermediates for cell growth, is induced by certain oncogenes [23]. The present studies demonstrate that transformation of rat 3Y1 fibroblasts with the MUC1-C oncoprotein is associated with increased uptake of glucose and production of lactate. Moreover, silencing of MUC1-C in human breast cancer cells resulted in decreased glucose uptake and lactate production, indicating that MUC1-C contributes to aerobic glycolysis. Activation of the PI3K->Akt pathway has been linked to glucose metabolism through glucose transporter expression, and stimulation of hexokinase and phosphofructokinase activities [24]. In that sense, MUC1-C activates the PI3K->Akt pathway, at least in part, through direct binding of PI3K to a consensus pYHPM motif in the MUC1-C cytoplasmic domain [18], [19]. Thus, MUC1-C could promote aerobic glycolysis through activation of PI3K->Akt signaling. Indeed, silencing Akt in 3Y1/MUC1-CD cells was associated with decreased glucose uptake and partial suppression of lactate production, indicating that MUC1-CD may regulate aerobic glycolysis by Akt-dependent and –independent mechanisms. The present studies provide another link between MUC1-C and aerobic glycolysis through interactions with PKM2. The PKM2 isoform is necessary for conferring the Warburg effect and enables cancer cells to divert glucose metabolites for the synthesis of macromolecules in the response to growth factor stimulation [4], [6], [22]. In this capacity, the regulation of PKM2 is of importance to the metabolism of cancer cells and their ability to form tumors [6]. However, little is known about proteins that interact with PKM2 and affect its activity.

### MUC1-C subunit associates with PKM2

Our studies demonstrate that MUC1-C binds directly to the PKM2 B- and C-domains. A cysteine in the MUC1-C cytoplasmic domain, designated Cys-3, interacts with the PKM2 B-domain Cys-165, which is highly conserved in the other PK isoforms and resides on the PKM2 surface near the hinge region that dictates the size of the active site cleft [7] (structural model shown in Fig. S2). To our knowledge, there are no other proteins that are known to bind to the PKM2 B-domain. Mutation of Cys-165 had no effect on basal or MUC1-CD Cys-3-induced PKM2 activity; thus, further study will be needed to define the significance of this MUC1-C interaction with the PKM2 B-domain. In this context, the MUC1-C cytoplasmic domain forms complexes with c-Src and c-Abl, and thereby could function as an adapter that facilitates the association of PKM2 with these tyrosine kinases [11]. By extension, c-Src-mediated tyrosine phosphorylation is associated with inhibition of PKM2 activity [8]. Our results also demonstrate that the MUC1-C cytoplasmic domain Cys-3 binds to the PKM2 C-domain at Cys-474. The PKM2 Cys-474 residue is found in the M1, but not the R or L, isoform and is conserved among other mammalian species. PKM1 and PKM2 differ by the presence in PKM2 of a 56 aa region (aa 378–434) that forms the FBP-binding allosteric pocket [7]. PKM2 Cys-474 thus resides outside the FBP-binding site and the inhibitory Lys-433 residue [8]. Nonetheless, PKM2 Cys-474 is positioned on the PKM2 surface (Fig. S2). Other work has shown that the Oct-4 transcription factor binds to the PKM2 C-domain; however, the effect of that interaction on PKM2 activity was not assessed in those studies [25]. Recent studies have also shown that PKM2 also interacts with HIF-1α in the absence of an effect on PKM2 enzymatic activity and is hydroxylated by PHD3 at Pro-403 in the C-domain [26]. Our findings lend support to the importance of the PKM2 Cys-474 residue in protein binding and, in turn, the regulation of PKM2 activity.

### MUC1-C cytoplasmic domain positively and negatively regulates PKM2 activity

The present results show that direct binding of MUC1-CD to recombinant PKM2 in vitro increases PKM2 activity. Addition of FBP to recombinant PKM2 stimulated PKM2 activity and MUC1-CD induced an additive increase. To search for supportive evidence, we incubated PKM2 with a MUC1-CD-derived peptide, designated GO-203, that contains the MUC1-CD Cys-3 residue. As found with MUC1-CD, GO-203 stimulated PKM2 activity and this effect was additive with FBP. Notably, GO-203-induced stimulation of PKM2 activity was abrogated by mutation of PKM2 Cys-474, indicating that the direct interaction with this site is responsible for PKM2 activation. Conversely, the present results demonstrate that MUC1-CD also functions as a negative regulator of PKM2 activity. Phosphorylation of PKM2 on Tyr-105 by fibroblast growth factor receptor type 1 is associated with inhibition of PKM2 activity [9]. Bcr-Abl, JAK2 and FLT3 also downregulate PKM2 by phosphorylation of the Tyr-105 site [9]. Our results in rat fibroblasts and in human breast cancer cells demonstrate that MUC1-C has no effect on PKM2 Tyr-105 phosphorylation. Other work has shown that binding of phosphotyrosine peptides to PKM2 Lys-433 results in release of FBP and thereby inhibition of PKM2 activity [8]. The MUC1-C cytoplasmic domain contains a YEKV motif that is phosphorylated by EGFR [21], [27] and corresponds to phosphotyrosine peptides that inhibit PKM2 [8]. For that reason, we asked if EGFR-phosphorylated MUC1-CD binds to the PKM2 C-domain Lys-433. To directly address this possibility, we mutated MUC1-CD Cys-3 to block binding to the PKM2 C-domain Cys-474. Under these experimental conditions, EGFR-phosphorylated MUC1-CD(C3A) exhibited specific binding to the PKM2 C-domain that was attenuated by mutation of Lys-433. EGFR-phosphorylated MUC1-CD(C3A) also inhibited PKM2 activity. As further evidence, a smaller phospho-YEKV peptide devoid of the Cys-3 residue similarly inhibited PKM2 by a Lys-433-dependent mechanism. In concert with previous results [8], binding of EGFR-phosphorylated MUC1-CD(C3A) or the phospho-YEKV peptide to PKM2 blocked FBP-induced activity. By contrast, activation of PKM2 by GO-203 binding to Cys-474 was unaffected by the phospho-YEKV peptide. These findings indicate that MUC1-CD activates PKM2 through the Cys-474 site and inhibits PKM2 by phosphotyrosine peptide binding to Lys-433.

### Why would MUC1-C play a role in the regulation of aerobic glycolysis and PKM2 activity?

The present studies further indicate that MUC1-C stimulates or suppresses PKM2 activity in breast cancer cells by a mechanism associated with tyrosine phosphorylation of the MUC1-C cytoplasmic domain. In this context, EGF stimulation induced phosphorylation of the MUC1-C cytoplasmic domain on tyrosine and suppressed PKM2 activity by a MUC1-C-dependent mechanism. These results are in concert with inhibition of PKM2 activity by binding of the EGFR-phosphorylated MUC1-C cytoplasmic domain. Nonetheless, given the diversity of signals that are induced by EGFR activation, including regulation of the pentose phosphate pathway in glycolysis, these results do not exclude the possibility that MUC1-C suppresses PKM2 activity by another mechanism. Arguably, as noted above, MUC1-C could bind to the PKM2 B-domain Cys-165 and function as an adapter to facilitate an interaction between c-Src and PKM2. In this regard, phosphorylation of the MUC1-C cytoplasmic domain on the YEKV motif in turn functions as a binding site for the c-Src SH2 domain [27]. The available evidence indicates that MUC1-C functions physiologically in signaling stress at the apical border of normal epithelial cells that interface with the external environment [11]. In the epithelial stress response with reversible loss of polarity, apical cell surface proteins, such as MUC1-C, can transiently form complexes with activated receptor tyrosine kinases to promote proliferation and repair of the epithelial layer [11]. In carcinoma cells with irreversible loss of polarity, the MUC1-C subunit constitutively interacts with EGFR and promotes EGFR signaling [12]. Therefore, it is formally possible that an EGFR-mediated proliferative response involving overexpression of the MUC1-C subunit has been appropriated and subverted by cancer cells to promote their own growth and survival. The overexpression of MUC1-C is sufficient to induce transformation [13], [14] and resistance to cell death [28], [29], [30], [31]. Cancer cells could thus conceivably exploit MUC1-C-mediated regulation of aerobic glycolysis and PKM2 to divert glycolytic metabolites for tumor cell growth and survival. Alternatively, it is also conceivable that binding of MUC1-C to the PKM2 Cys-474 may under certain metabolic conditions be functionally important in activating PKM2 for the increased production of pyruvate and thereby (i) ATP through the TCA cycle, and (ii) acetyl-CoA and lipid synthesis. For example, the redox state of the cancer cell could affect reactivity of the MUC1-C Cys-3 residue and thereby dictate whether MUC1-C binds to PKM2 Cys-474 and induces PKM2 activity. Thus, further studies will be needed that address the effects of MUC1-C on the pentose phosphate pathway, glucose-derived lipid biosynthesis, oxygen consumption and ATP production in cancer cells. In summary, the present findings demonstrate that MUC1-C stimulates aerobic glycolysis and that this response may be mediated, at least in part, by both activation of the Akt pathway and interactions with PKM2.

## Materials and Methods

### Cell culture

Rat 3Y1/vector and 3Y1/MUC1-CD cells [14] were cultured in DMEM medium containing 10% heat-inactivated fetal bovine serum (FBS), 100 units/ml penicillin, 100 µg/ml streptomycin and 2 mM L-glutamine. Human ZR-75-1, MCF-7 (ATCC, Manassas, VA), ZR-75-1/vector, ZR-75-1/MUC1siRNA [32], MCF-7/vector and MCF-7/MUC1siRNA [33] breast cancer cells were grown in RPMI1640 medium with 10% FBS, antibiotics and L-glutamine. Human HCT116 colon cancer cells were cultured in Dulbecco's modified Eagle's medium with 10% FBS and antibiotics [20]. Cells were transiently transfected with control, Akt siRNA and MUC1 siRNA pools (Dharmacon, Lafayette, CO) in the presence of Lipofectamine (Invitrogen, Carlsbad, CA). In certain studies, cells were cultured with 0.1% FBS for 24 h and then stimulated with 10 ng/ml EGF (Sigma, St. Louis, MO).

### Glucose uptake and lactate production

Cells (10^5^/60 mm culture dish) were seeded for 24 h and the culture supernatant was then replaced with 5 ml of FBS-free and L-glutamine-free medium. After incubation for 8 h, the supernatant was collected, centrifuged and stored at −80°C. Glucose uptake was measured using the Amplex Red Glucose/Glucose Oxidase Assay Kit (Molecular Probes, Carlsbad, CA). Lactate production was measured by the Lactate Assay Kit (BioVision, Mountain View, CA).

### Pyruvate kinase assay

Pyruvate kinase activity was measured using the Pyruvate Kinase Assay Kit (BioVision). The assays were performed with 1 µg of cell lysate prepared as described [29]. Alternatively, 5 µM recombinant His-PKM2 was incubated in the absence and presence of MUC1-CD, FBP (Sigma), GO-203, CP-2, TDRSPpYEKVSA or TDRSPYEKVSA peptide for 30 min at room temperature and then assayed for activity. The results were determined from three to five separate experiments each performed in triplicate.

### Immunoprecipitation and immunoblotting

Lysates from subconfluent cells were prepared as described [29]. Soluble proteins were precipitated with anti-PKM2 (Cell Signaling Technology, Danvers, MA) and anti-MUC1-C (Ab5; NeoMarkers, Fremont, CA). The precipitates and cell lysates were immunoblotted with anti-MUC1-C, anti-PKM2, anti-p-PKM2(Tyr-105), anti-p-Tyr (Cell Signaling Technology) and anti-β-actin (Sigma). Immune complexes were detected with horseradish peroxidase-conjugated secondary antibodies and enhanced chemiluminescence (GE Healthcare Biosciences, Salt Lake City, UT).

### In vitro binding assays

Human PKM2 was cloned into pET28a (EMD Chemicals, Gibbstown, NJ) to express an N-terminal His-tagged fusion protein. Recombinant PKM2 and PKM2 deletion mutants were purified from E. coli using Ni-agarose beads (Qiagen, Valencia, CA). GST, GST-MUC1-CD, GST-MUC1-CD(1-45), GST-MUC1-CD(46-72) and GST-MUC1-CD with specific point mutations were prepared as described [32]. Purified GST-MUC1-CD proteins bound to glutathione beads were incubated with 10 units EGFR (Sigma) in the presence of 200 µM ATP for 30 min at 30°C. Adsorbates to glutathione-conjugated beads were analyzed by immunoblotting. In certain experiments, phosphorylated GST-MUC1-CD was cleaved with thrombin to remove the GST moiety.

### Colony formation assays

Soft agar colony formation assays were performed in medium containing 25 mM glucose supplemented with 10% FBS. Six-well plates were coated with 1.5 ml of 0.6% base medium agar. Cells were plated at a density of 1×10^4^ cells/well with 1.5 ml of 0.35% top medium agar. The cultures were incubated for 14 days. Colonies were stained with crystal violet and counted.

### Confocal microscopy

3Y1/vector and 3Y1/MUC1-CD cells were cultured on glass coverslips in 60 mm dishes. For staining mitochondria, the cells were incubated in serum-free medium containing 100 nM of MitoTracker Red CMXRos (Molecular Probes, Eugene, OR) for 30 min at 37°C. The cells were then washed with complete medium containing serum, pre-fixed with 3.7% formaldehyde for 15 min at 37°C, and permeabilized in 1% bovine serum albumin (BSA)–supplemented PBS containing 0.2% Triton X-100 for 5 min at 25°C. Post-fixation was done in 3.7% formaldehyde/PBS for 5 min at 25°C. The fixed cells were washed in PBS and then blocked with a mixture of 2% BSA and 5% nonfat milk for 1 hour at 25°C. The fixed and blocked cells were incubated with anti–PKM2 antibody (Abcam) overnight at 4°C, followed by incubation with ‘Alexa-Fluor 647’ anti-rabbit IgG secondary antibody (Invitrogen). Nuclei were stained with 4′, 6-diamidino-2-phenylindole (DAPI, 1 mg/ml; Invitrogen). After mounting the coverslips, images were captured with a Yokogawa spinning disk confocal microscope.

## Supporting Information

Figure S1 **Subcellular distribution of PKM2 in 3Y1/vector and 3Y1/MUC1-CD cells.** Confocal microscopy of 3Y1/vector and 3Y1/MUC1-CD cells stained with anti-PKM2 antibody. Mitochondria were stained with MitoTracker Red. Nuclei were stained with DAPI.
(TIF)

Figure S2 **Structural model for PKM2 highlighting the B-domain Cys-165 and C-domain Cys-474 sites.** View of the PKM2 monomer structure with the colored A- (blue), B- (green), C- (pink) and D- (brown) domains. Residues Cys-165, Cys-474 and Lys-433 are presented in a space-filling model.
(TIF)
