# Supplementary figures and images for: MUC1-C Oncoprotein Regulates Glycolysis and Pyruvate Kinase m2 Activity in Cancer Cells
Source: PLoS One. 2011 Nov 28;6(11):e28234. doi: 10.1371/journal.pone.0028234 (PMC3225393; doi:10.1371/journal.pone.0028234)

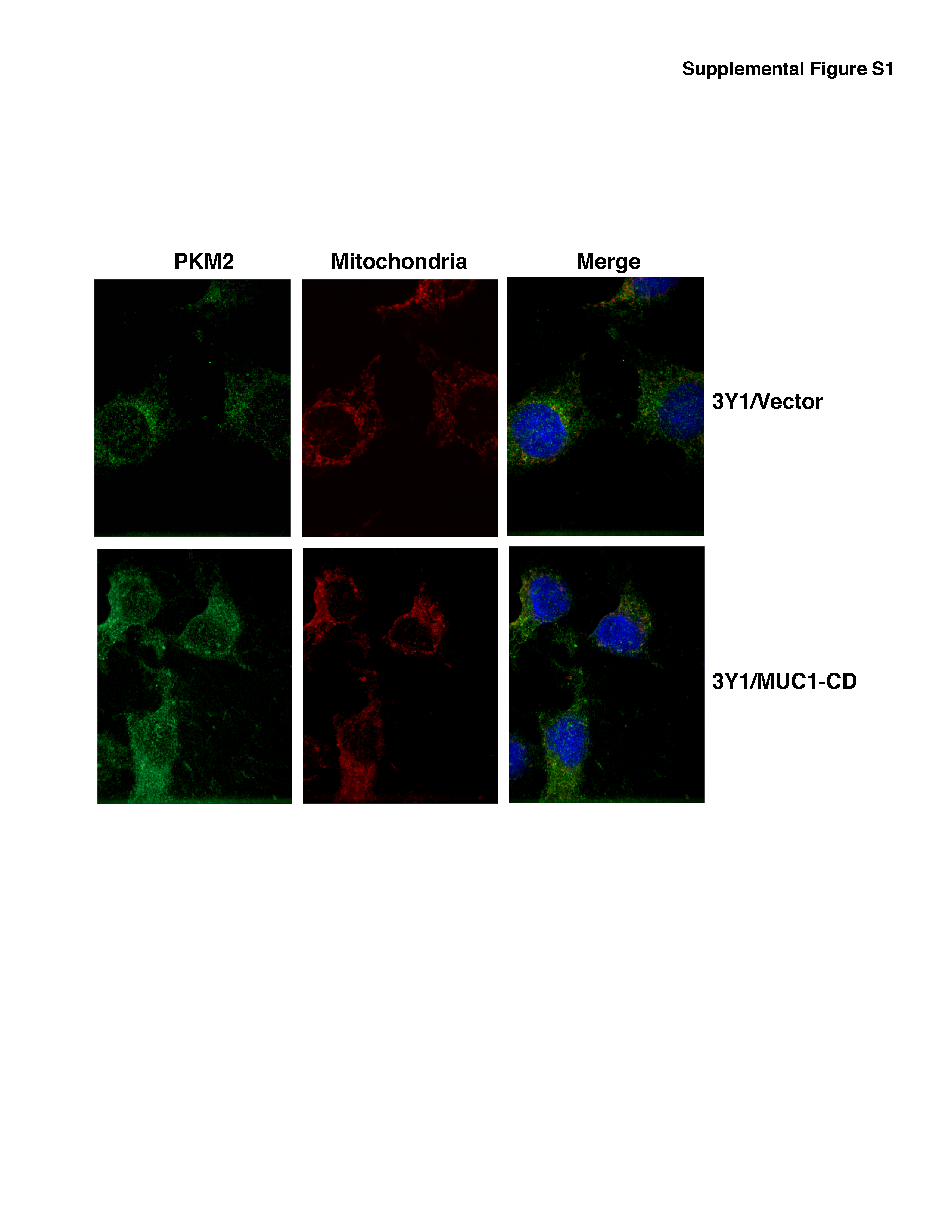

Supplement: Figure S1 — Subcellular distribution of PKM2 in 3Y1/vector and 3Y1/MUC1-CD cells. Confocal microscopy of 3Y1/vector and 3Y1/MUC1-CD cells stained with anti-PKM2 antibody. Mitochondria were stained with MitoTracker Red. Nuclei were stained with DAPI. (TIF) [file pone.0028234.s001.tif]

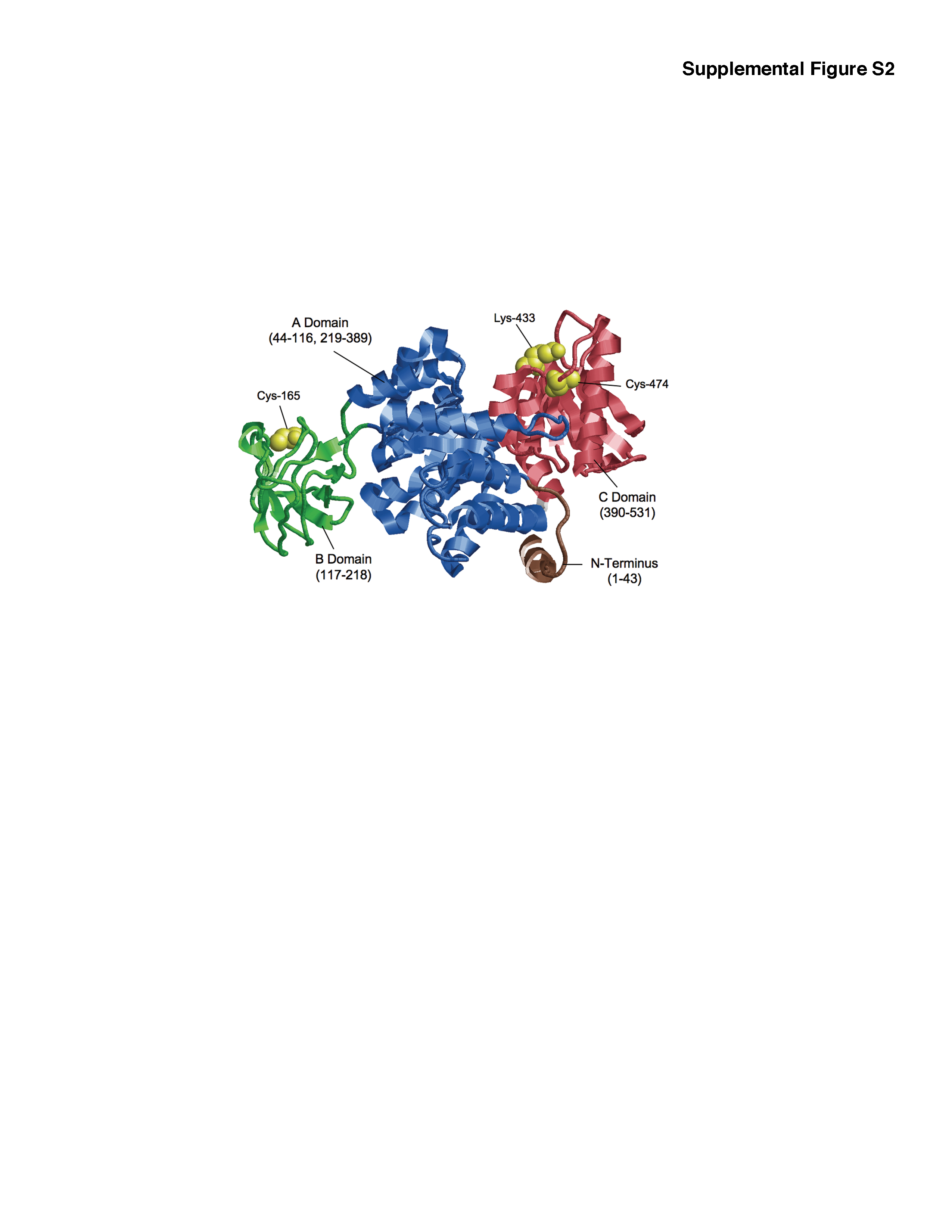

Supplement: Figure S2 — Structural model for PKM2 highlighting the B-domain Cys-165 and C-domain Cys-474 sites. View of the PKM2 monomer structure with the colored A- (blue), B- (green), C- (pink) and D- (brown) domains. Residues Cys-165, Cys-474 and Lys-433 are presented in a space-filling model. (TIF) [file pone.0028234.s002.tif]
